# Simulation-Optimization for Conjunctive Water Resources Management and Optimal Crop Planning in Kushabhadra-Bhargavi River Delta of Eastern India

**DOI:** 10.3390/ijerph17103521

**Published:** 2020-05-18

**Authors:** Madan K. Jha, Richard C. Peralta, Sasmita Sahoo

**Affiliations:** 1 Agricultural & Food Engineering Department, Indian Institute of Technology Kharagpur, Kharagpur-721 302, India; sasmitaiit@gmail.com; 2 Civil and Environmental Engineering, Utah State University, Logan, UT 84322-4110, USA; peralta.rc@gmail.com

**Keywords:** simulation-optimization model, integrated water resources management, optimal cropping pattern, linear programming, deltaic aquifer, India

## Abstract

Water resources sustainability is a worldwide concern because of climate variability, growing population, and excessive groundwater exploitation in order to meet freshwater demand. Addressing these conflicting challenges sometimes can be aided by using both simulation and mathematical optimization tools. This study combines a groundwater-flow simulation model and two optimization models to develop optimal reconnaissance-level water management strategies. For a given set of hydrologic and management constraints, both of the optimization models are applied to part of the Mahanadi River basin groundwater system, which is an important source of water supply in Odisha State, India. The first optimization model employs a calibrated groundwater simulation model (MODFLOW-2005, the U.S. Geological Survey modular ground-water model) within the Simulation-Optimization MOdeling System (SOMOS) module number 1 (SOMO1) to estimate maximum permissible groundwater extraction, subject to suitable constraints that protect the aquifer from seawater intrusion. The second optimization model uses linear programming optimization to: (a) optimize conjunctive allocation of surface water and groundwater and (b) to determine a cropping pattern that maximizes net annual returns from crop yields, without causing seawater intrusion. Together, the optimization models consider the weather seasons, and the suitability and variability of existing cultivable land, crops, and the hydrogeologic system better than the models that do not employ the distributed maximum groundwater pumping rates that will not induce seawater intrusion. The optimization outcomes suggest that minimizing agricultural rice cultivation (especially during the non-monsoon season) and increasing crop diversification would improve farmers’ livelihoods and aid sustainable use of water resources.

## 1. Introduction

Rapid population growth and climate change are likely to increase the risk of water shortage. Changing seasonal patterns of water availability will adversely impact social and economic development [1]. Water-energy-food system complexity, uncertain future climatic events, unsustainable abstraction of groundwater, and contamination from different sources pose challenges to resource planners and managers [2,3]. Coping with these challenges and strengthening water security require an adaptive integrated framework that endorses and facilitates coordinated development and the management of water, land, and related resources [4,5,6].

Scientific planning involves the ability to predict system response without, and with, management changes. Numerical simulation modeling is most commonly used to predict the dynamics and states of heterogeneous aquifer systems [7,8,9,10]. Using simulation-optimization (S-O) models that include influential physical or decision-making constraints and goals can help in selecting appropriate management strategies [11,12,13].

The knowledge that mathematical optimization yields better solutions to well-formulated optimization problems than does a trial-and-error approach, is so widespread that S-O modelling has been extensively employed for designing optimal water systems and strategies [14]. Examples are contamination remediation [14], conjunctive use of surface water and groundwater [14,15,16,17], irrigation management [18,19], crop planning [20,21], seawater intrusion management [22,23], groundwater-pumping optimization [24,25,26], drought analysis [27], climate impacts assessment [28], management of total suspended solids [29], and aquifer recharge management [30].

Embedded within some of the above hydraulic S-O models were physically based finite numerical flow equations or full numerical simulation models in order to represent hydraulic response to hydraulic stimuli during the optimization process. When used for large physical systems, such embedding approach S-O models addressed relatively large spatial discretization and few periods of steady boundary conditions [31,32,33].

An alternative to the embedding approach is the use of surrogate simulators as substitute hydraulic simulators during optimization [17,25,34,35,36,37,38,39]. The intent of using surrogate simulators is to replace an original complex simulation model by a simple and computationally inexpensive model during the optimization process. The response matrix method flexibly handles groundwater optimization problems of varying scales, and is widely used [12,40,41,42], especially for confined aquifers by using influence coefficients and discretized convolution equations as surrogate simulators. Refs. [14,26,34] describe the procedure for adapting a response matrix approach to accurately simulate nonlinear unconfined groundwater flow.

Classical optimization methods that are used in planning the management of surface water and groundwater resources include: linear programming [43,44,45], non-linear programming [46,47], dynamic programming [48,49], and hierarchical or multilevel optimization [50,51]. Systems engineering textbooks and ref. [14] provide examples that are used for groundwater management. For linear or linearizable management problems, linear programming (LP) requires the least computational expense and calculation time to compute globally optimal solutions. Heuristic or evolutionary algorithms (EA) and other metaheuristics are preferred for solving nonlinear, non-convex, and discrete water problems for which deterministic search techniques fail [52,53,54,55]. Among the EAs, Genetic Algorithm (GA) has been most commonly applied for water problems [56,57,58]. Ref. [38] used multi-objective GAs for the conjunctive use of reservoir, stream, and groundwater resources. Ref. [59] used GA and dynamic programming optimization for multi-purpose water management. Water systems engineering textbooks and Ref. [14] compare application of alternative optimization techniques for water management problems. Ref. [14] shows that using the response matrix with LP is much more efficient than using heuristic optimization and fully embedded flow simulation models.

The standard systems optimization procedure is to use the simplest and computationally least costly approach that will yield the best solution for the problem. Widely available LP algorithms are guaranteed to compute the best solutions for linear optimization problems. LP optimization is used here to perform the reconnaissance evaluation.

The study goal is to propose and demonstrate a simulation–optimization framework for integrated water resources management in a deltaic river basin near the coast in Eastern India. First, the proposed groundwater management model incorporates a calibrated and validated groundwater flow simulation model (MODFLOW-2005: USGS Three-Dimensional Finite-Difference Groundwater Model, [60]) within the Simulation-Optimization MOdeling System (SOMOS, [34]) module number 1 (SOMO1, [34]) to determine maximum simultaneous extraction rates from all existing irrigation wells, subject to constraints for management and aquifer protection from seawater intrusion. The distributed maximum pumping rates that will not cause salt water intrusion are used as the upper limits on groundwater extraction in the next model. The second optimization model conjunctively allocates surface water, groundwater, and land to develop a cropping pattern that maximizes net annual returns from crop yield, without causing salt water intrusion. The second model uses linear programming optimization while considering the types of climatic seasons, suitability of available cultivable land, existing basin crops’ unit costs and benefits, and the hydrogeology-based groundwater pumping constraints. The applicability and efficacy of the proposed S-O approach are demonstrated for a coastal deltaic aquifer system.

## 2. Methodology

### 2.1. Study Area and Hydro-Climatic Settings

**Figure 1:**
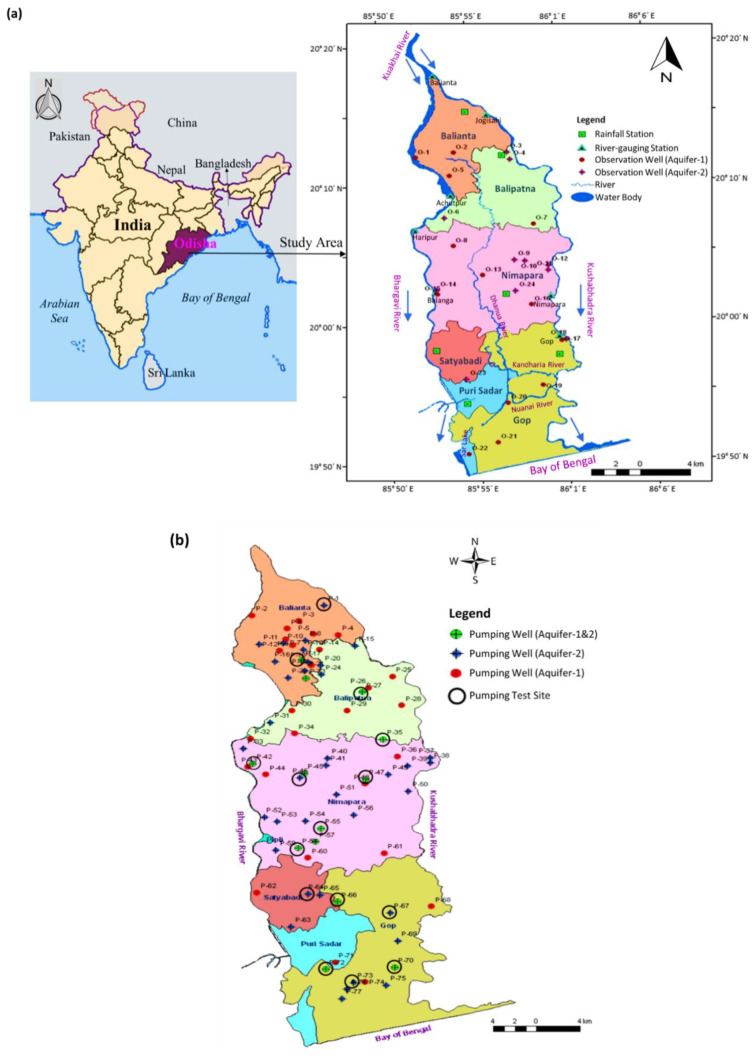
(**a**) Location of the study area with rainfall stations, river gauging stations and observation wells; and (**b**) pumping wells in the unconfined aquifer (Aquifer-1) and confined aquifer (Aquifer-2) (modified from [62]).

The target study area is the Kushabhadra-Bhargavi River Delta, bounded by the Kushabhadra River and Bhargavi River (Figure 1a). It lies upstream of the Kuakhai River within the Mahanadi Delta region of Odisha, Eastern India. The topographic elevation of the 620-km^2^ area varies from 0 to 26 m MSL (above Mean Sea Level). The area has a tropical monsoon climate. Three distinct seasons prevail in the study area: monsoon (rainy), winter, and summer. The monsoon (rainy) season spans from mid-June to October-end. The winter season starts from November and lasts until the end of February, and the summer season extends from March to mid-June. From the agricultural point of view, the year can be divided into three cropping seasons, viz. Kharif, Rabi, and Summer. The average annual rainfall in the study area is about 1416 mm with a majority of rainfall occurring in the monsoon season from the southwest monsoon. The mean monthly maximum and minimum temperatures in the area are 42 °C in the month of May and 17 °C in the month of December.

Canal water and groundwater generally irrigate approximately 40% and 60% of the total study area, respectively. However, more groundwater is used in non-monsoon periods or in the years having less than the normal rainfall. The surficial laterite and alluvium are underlain by an unconfined aquifer at shallow depths and a confined aquifer at greater depths [61]. The water table in the unconfined aquifer ranges from 1.02 to 8.74 m bgs (below the ground surface) in the pre-monsoon season to 0.98 to 7.14 m bgs in the post-monsoon season. The piezometric level of the confined aquifer varies between 0.68 and 9.05 m bgs before the monsoon season and 0.18 to 8 m bgs after the monsoon season. The freshwater aquifers are appreciably recharged during the monsoon season [62]. Ref. [63] used lithology and hydrogeologic data from the Orissa Lift Irrigation Corporation to build a conceptual model of the study area (Kushabhadra-Bhargavi interbasin).

### 2.2. Integrated Simulation-Optimization Framework

#### 2.2.1. Overview

The presented integrated Simulation-Optimization methodology involves three computer models. First, to enable predicting system response to changes in groundwater extraction is a Groundwater Simulation Model (GSM). GSM provides the initial aquifer heads that exist, and the ability to quantify head response to abstraction. Working with GSM, the Simulation-Optimization Groundwater Model (S-OGM) develops surrogate simulators of head response to pumping, and then computes the spatially distributed maximum groundwater extraction rates that will not cause saltwater intrusion from the ocean. The soil and water Resource Optimization Module (ROM) uses those extraction rates as the upper limits on groundwater abstraction while computing optimal cropping patterns and strategies for the conjunctive of groundwater and surface water. Subsequent sub-sections provide more details.

#### 2.2.2. Groundwater Simulation Model (GSM)

**Figure 2:**
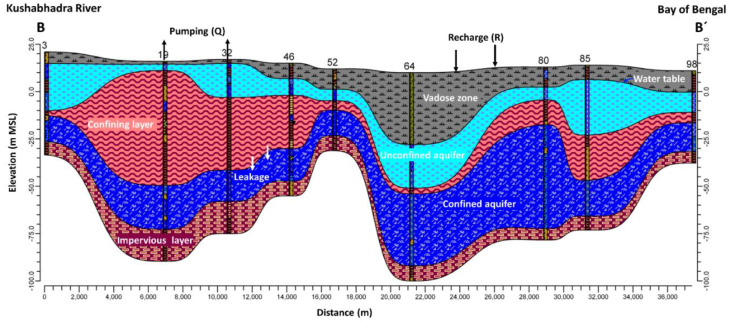
Conceptual model of the study area at B-B’ cross-section (adapted from [62]).

Figure 2 depicts a cross section of the study area. To simulate flow, this study employed the calibrated MODFLOW-2005 groundwater simulation model reported previously [62]. The top-most aquifer layer (Aquifer-1) is unconfined and underlain by a leaky confining layer. Immediately below the confining layer is the confined Aquifer-2 layer, the major source of groundwater in the area. Aquifer-2 has thickness varying from 3.1 to 80.3 m and is underlain by impermeable clay or bedrock.

Initial estimates of aquifer parameter values came from pumping-test data of 15 sites [64], and other sources [62]. Model calibration and validation targets included observed groundwater-levels at 24 sites from 1997 to 2006, and 2007–2011, respectively Ref. [62]. The resulting calibrated and validated parameters are the hydraulic conductivity of Aquifer-1 and Aquifer-2, vertical hydraulic conductivity of the leaky confining layer, specific yield of Aquifer-1, storativity of Aquifer-2, groundwater pumping, and aquifer recharge. Ref. [62] provides details of model calibration, validation, and sensitivity analysis.

This MODFLOW-2005 implementation enabled the prediction of aquifer head response to groundwater abstraction. The MODFLOW-2005 model supported the development of Optimization Model-1 discussed next.

#### 2.2.3. Simulation-Optimization Groundwater Model (S-OGM)

**Figure 3:**
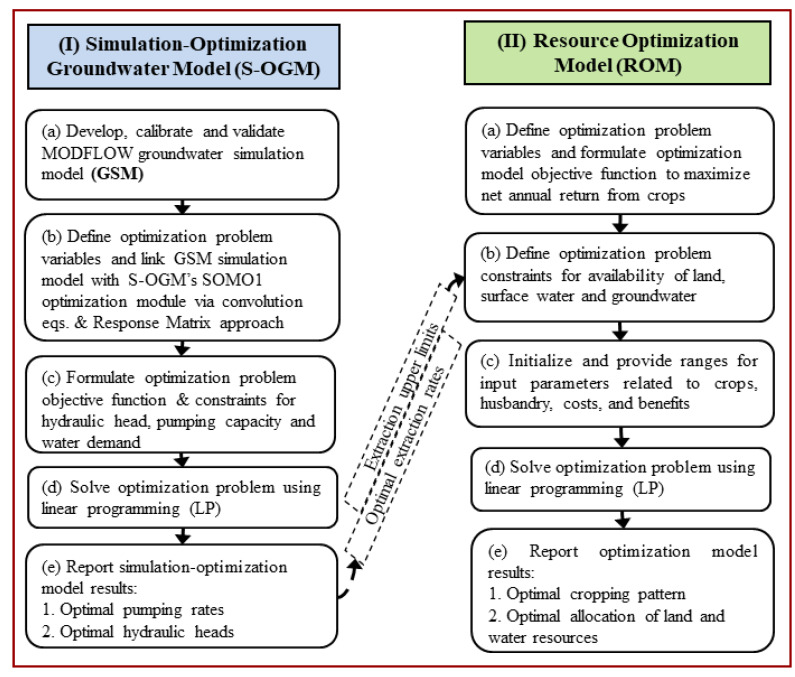
Schematic representation of integrated modeling framework for optimal land and water resources allocation to crops: (I) Simulation-Optimization Groundwater Model (S-OGM) for computing optimal groundwater abstraction strategies; and (II) Resources Optimization Model (ROM) for determining optimal cropping patterns and land and water resources allocations.

The goal of this model was to mathematically determine, for agriculturally productive weather seasons, sets of spatially distributed simultaneous maximum groundwater pumping rates that could be extracted from existing wells without causing undesirable consequences. Figure 3 shows the general process that is involved in computing such a set of maximum simultaneous pumping rates. **Step (Ia)** is preparation and validation of the previously described finite difference groundwater-flow model (MODFLOW-2005) for the study area. **Step (Ib)** defines decision variables (groundwater pumping extraction rates for each well and time period, and a group of decision variables), and state variables (groundwater heads at selected aquifer control locations for each period). The SOMO1 module of the SOMOS software maps simulator inputs and outputs to and from an optimization algorithm (the Optimizer). SOMO1 also develops surrogate simulation equations that can compute groundwater heads that result from a set of pumping rates. These surrogate simulators are discretized convolution equations consisting of pumping rates and influence coefficients (a Response Matrix is an array of such coefficients), as detailed in Section 2.2.3.1. During subsequent mathematical optimization, the surrogates are used instead of the MODFLOW-2005 simulator to relate pumping rates to heads. In **Step (Ic)**, inputs to SOMO1 define the optimization problem objective function and constraints (including upper and lower bounds on state variables, and individual and groups of decision variables). **Step (Id)** invokes the Optimizer that solves the optimization problem. Finally, **Step (Ie)** reports the optimal pumping strategy and resulting heads.

The below Section 2.2.3.1 illustrates a convolution equation, such as is formed by **Step (Ib)** of Figure 3. Subsequent Section 2.2.3.2 describes the mathematical optimization problem that S-OGM solves.

##### 2.2.3.1. Response Matrix Approach

In groundwater simulation, a Response Matrix is an array of influence coefficients, each of which describes how a particular state variable will respond to a specific hydraulic stimulus. Each row of a Response Matrix has the coefficients of the discretized convolution equation that estimate one state variable’s value. For example, Equation (1) computes hydraulic head *h* at one location $\hat{o}$ and time *n* in response to initial and background conditions and the pumping from all decision variables until that time [14,65,66]. Each convolution equation is a substitute or surrogate simulator that accurately predicts the groundwater head in confined aquifers [34]. Refs. [14,63] provide details of adapting use of such equations within SOMO1 for application to nonlinear unconfined aquifers.

(1) $$h_{\hat{o},n}=h_{{}_{\hat{o},n}}^{i}+\sum_{k=1}^{n}\sum_{\hat{u}=1}^{M}\partial_{\hat{o},\hat{u},n-k+1}^{h}\frac{p_{\hat{u},k}}{p_{\hat{u}}^{ut}}$$

where, *h* = hydraulic head (m MSL) at location $\hat{o}$ at the end of time period *n*; $\hat{o}$ = index denoting an observation location at which system response is being evaluated; *n* = number of stress periods up to the time for which head is computed; *h^i^* = background head that will exist at specified location and time if no pumping occurs at the M candidate wells (m MSL); *k* = index for stress period; *M* = total number of candidate wells at which groundwater can potentially be pumped from the aquifer; $\hat{u}$ = index denoting a candidate pumping well location; $\partial_{\hat{o},\hat{u},n-k+1}^{h}$ = influence coefficient describing the effect of a unit of groundwater pumping at pumping well location $\hat{u}$ in stress period *k* on the hydraulic head at location $\hat{o}$ by the end of time period *n*; *p_û_*,*_k_* = pumping rate (m^3^/day) at pumping well location $\hat{u}$ during stress period *k*; and, $p_{\hat{u}}^{ut}$ = magnitude of unit pumping stimulus (m^3^/day) in pumping well location $\hat{u}$ that occurs during period one. SOMO1 used the calibrated MODFLOW-2005 to develop transient influence coefficients $\partial_{\hat{o},\hat{u},n-k+1}^{h}$ by subjecting each candidate pumping well to a unit-pumping stimulus. SOMO1 was used within the Simulation-Optimization Groundwater Model (S-OGM) described next.

##### 2.2.3.2. Formulation of Simulation-Optimization Groundwater Model (S-OGM)

S-OGM, a Simulation-Optimization Groundwater Model, is designed to determine, for assumed initial and boundary conditions, a set of maximum simultaneous physically feasible extraction rates from existing pumping wells that will at least satisfy historic groundwater need, without permitting seawater intrusion from the ocean. The groundwater management model determined simultaneous optimal abstractions from 77 pumping wells [31 tapping unconfined aquifer (Aquifer-1) and 46 tapping confined aquifer (Aquifer-2)]. Over the study area S-OGM for two seasons (monsoon season and non-monsoon season) per year for the 1997–2011 period while ensuring that the resulting heads at 24 sites, pumping rates of individual wells, and total pumping constraints described below are satisfied. The objective function of S-OGM is expressed as:

(2) $$Maximize\;Q_{k}=\sum_{\hat{u}=1}^{77}C_{k}^{p}p_{\hat{u},k}\;\mathrm{for}\;k=1,\;2\;\mathrm{for}\;\mathrm{each}\;\mathrm{year}$$

where, *Q_k_* = total groundwater extraction (m^3^) from 77 existing pumping wells during the *k*th stress period (*k* = 1 for monsoon period, and *k* = 2 for non-monsoon period); $p_{\hat{u},k}$ = pumping rate (m^3^/day) of the pumping well $\hat{u}$ during the *k*th stress period; and, $C_{k}^{p}$ = coefficient to convert flow rate into volume.

Optimization was performed subject to the following constraints.

(i) Aquifer Head Constraint:

At 24 locations, aquifer head constraints establish lower and upper limits on aquifer hydraulic heads ($h_{\hat{o},k}$), at head control points, allowed to result from optimal pumping. In both aquifers, the lower bound of head was 1 m above the mean sea level to prevent seawater intrusion into the aquifer. The upper bound of head was 2 m below the ground surface.

(ii) Constraints on Individual Wells and Total Pumping Capacity:

The pumping in each well cannot be injection and groundwater abstraction cannot exceed the pumping capacity of a well. The lower bound of pumping was zero and the upper bound was the pumping capacity. The upper bound on total system-wide pumping extraction is the sum of 77 well capacities.

(iii) Water Demand Constraint:

The total pumping from the basin should satisfy the total groundwater needs of the crops grown in the study area. In this study, the right-hand side of Equation (3) is the average 1997–2006 seasonal irrigation water demand.

(3) $$\sum_{\hat{u}=1}^{77}C_{k}^{p}p_{\hat{u},k}\geq WD_{k}\;\mathrm{for}\;\hat{u}=1,\;2,\;3,\ldots \ldots ,\;77\;\mathrm{and}\;k=1,\;2\;\mathrm{for}\;\mathrm{each}\;\mathrm{year}$$

where, *WD_k_* = groundwater demand of the crops (m^3^) during the *k*th stress period (*k* = 1 for monsoon period and *k* = 2 for non-monsoon period). A SIMPLEX linear programming (LP) algorithm [67], available with GAMS is used here to compute the optimal solution to the above optimization problem [68].

#### 2.2.4. Formulation of Resource Optimization Model (ROM)

Figure 3 shows the procedures developed to determine optimal cropping patterns in *Kharif*, *Rabi*, and *Summer* cropping seasons by maximizing net annual returns subject to relevant constraints. The developed ROM code considered the conjunctive use of surface water and groundwater resources and efficiently utilized the cultivable land to produce crops, without abstracting so much groundwater that seawater would intrude into the aquifers. The data concerning existing seasonal crops, gross irrigation requirements, cost of cultivation for different crops, and annual income from different crops were obtained from the Directorate of Economics and Statistics, Odisha. Table 1 summarizes the area covered and gross irrigation requirement for the crops presently cultivated in the study area during *Kharif* season (mid-June to October-end), *Rabi* season (November to February-end), and *Summer* season (March to mid-June). It is apparent from this table that the type of crops grown in the study area and the areas covered by the crops vary considerably from one season to another. The gross irrigation requirements of the crops range from 0.36 to 0.48 m in the *Kharif* season, 0.18 to 2.04 m in the *Rabi* season, and 0.24 to 0.54 m in the *Summer* season. Further, the largest water requirements are for the sugarcane (2.04 m) and paddy (1.44 m) crops grown in the *Rabi* season.

The ROM resource management objective function was to maximize total net annual economic return:

(4) $$Max\;Z=\sum_{i=1}^{3}\sum_{c=1}^{26}(P_{c}Y_{i,c}-C_{c})A_{i,c}$$

where, *Z* = net total annual return (Rs.); *P_c_* = market price of the *c*th crop (Rs./kg); *Y_i_*_,*c*_ = yield of the *c*th crop (kg/ha) in the *i*th type of land (ha); *C_c_* = cost of cultivation including irrigation cost per unit area for the *c*th crop (Rs./ha); *A_i_*_,*c*_ = area under the *c*th crop in the *i*th type of land (ha); *i* = index denoting land type (1 for high land, 2 for medium land, and 3 for low land); and, *c* = index for crop type.

Optimization was performed using the following constraints.

(i) Constraints on Individual Well and Total Pumping Capacity:

The pumping capacity of each well was used as the upper limit for extraction at that well. The upper limit on total groundwater pumping is the sum of the individual well capacities.

(ii) Land Availability Constraint:

Only crops that were suitable for a particular land category (high, medium, and low) were available for assignment to that land category. In each season, the total land area assigned to all crops within cultivable land category *i*, cannot exceed *A_i_*, the total cultivable land of that category. Because two crops are suitable for the *Kharif* season and 24 additional crops are suitable for *Rabi* and *Summer* seasons, land availability constraints are expressed as:

For *Kharif* season:

(5) $$\sum_{c=1}^{2}A_{i,c}\leq A_{i}\;\mathrm{for}\;i=1,\;2,\;3$$

For *Rabi* and *Summer* seasons:

(6) $$\sum_{c=3}^{26}A_{i,c}\leq A_{i}\;\mathrm{for}\;i=1,\;2,\;3$$

where, *A_i_*_,*c*_ = area under the *c^th^* crop in the *i^th^* type of land (ha), *A_i_* = total cultivable area of the *i^th^* type of land (*i* = 1 for high land, *i* = 2 for medium land, and *i* = 3 for lowland), and *A_i_*_,*c*_ ≥ 0 *for* all *i* and *c*.

(iii) Water Availability Constraint

In each stress period, the total provided irrigation water cannot exceed the sum of groundwater pumped and SW provided during that stress period. This equals total maximum permissible groundwater extraction from all of the pumping wells plus the maximum surface water extraction from all the sources, such as river, canal, surface lifts, tank storages, etc. The water availability constraints are expressed as:

For Monsoon (*Kharif* season) period:

(7) $$\sum_{i=1}^{3}\sum_{c=1}^{2}W_{i,c}A_{i,c}\leq GW_{k=1}+SW_{k=1}.$$

For non-monsoon (*Rabi* and *Summer* seasons) period:

(8) $$\sum_{i=1}^{3}\sum_{c=3}^{26}W_{i,c}A_{i,c}\leq GW_{k=2}+SW_{k=2}$$

where, *A_i_*_,*c*_ = area under the *c^th^* crop in the *i^th^* type of land (ha), *W_i_*_,*c*_ = water requirement of the *c^th^* crop in the *i^th^* type of land (ha), $GW_{k}=\sum_{\hat{u}=1}^{77}C_{k}^{p}p_{\hat{u},k}$= maximum S-OGM groundwater extraction via all candidate wells in the stress period *k* = 1 (monsoon period) or *k* = 2 (non-monsoon period), and *SW_k_* = maximum surface water utilized for irrigation historically in the *k^th^* stress period.

For each of years 2004–2011, this optimization problem was solved using LP via the MATLAB optimization tool. Here, we discuss the optimization that utilized the information from 2009. That data included utilizable crops, irrigated crop yield, cultivation cost, including irrigation cost per unit area, net annual return, seasonal gross irrigation requirement per unit crop area, upper limit on total surface water diversion, and maximum permissible groundwater abstraction (from the Simulation-Optimization Groundwater Model). The LP model output was the maximum net annual benefit from the cultivated crops, and the associated optimum land and water utilization.

## 3. Results and Discussion

### 3.1. Relations between Rainfall, River Stage, and Groundwater Levels

**Figure 4:**
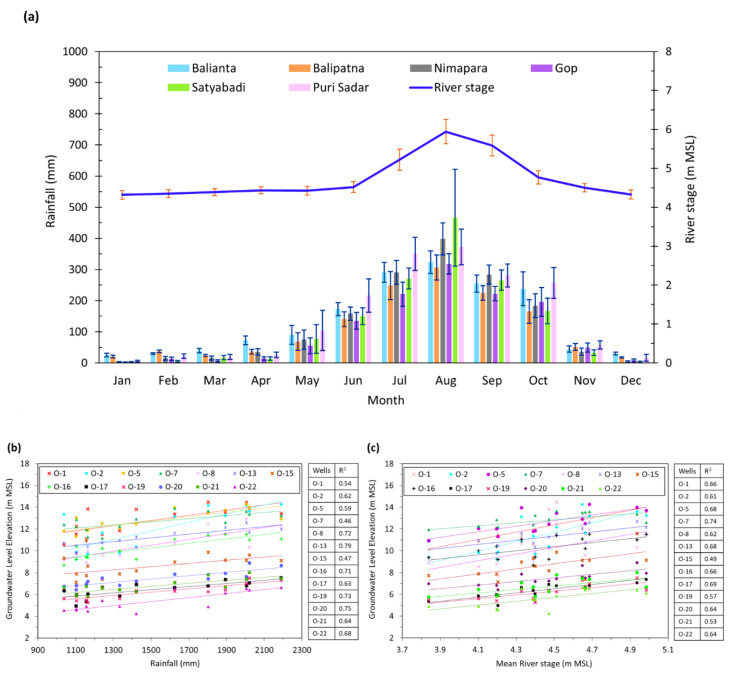
(**a**) Monthly rainfall and river stage patterns in the study area (adapted from [62]); and regression analyses (**b**) between rainfall and groundwater levels at 13 sites (observation wells) of Aquifer-1, and (**c**) between river stage and groundwater levels.

**Figure 5:**
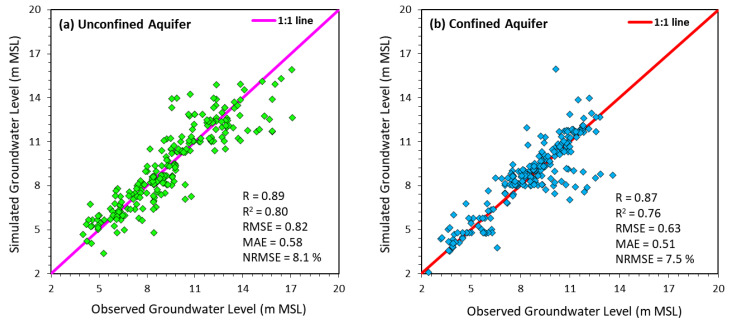
Scatter plots contrasting simulated and observed groundwater levels-in (**a**) unconfined aquifer and (**b**) confined aquifer during model validation period (modified from [62]).

Figure 4a shows the monthly rainfall depths in the study area over 21 years (1990–2010) at six rainfall stations. The rainy season spans from mid-June to the end of October. The greatest rainfall variation occurred from May to October, and the least variation occurred from November to April. Satyabadi station had the highest rainfall in August. Nimapara, Gop, and Satyabadi stations had less rainfall in December and January than the other three stations. Figure 5a also shows monthly variation in Kushabhadra River water stage at the Nimapara gauging station during 1990–2011. The stream stage varied significantly with time. The minimum river stage occurred during December–May when rainfall is lowest. The maximum river stages occurred during July–October, followed by June and November. The standard error bars show that the greatest river stage variability was in August, followed by July, September, October, and June. The river stage varied little from November to May.

Figure 4b shows that during the 14 years of 1997–2010, rainfall greatly influenced Aquifer-1 groundwater levels—the regression R^2^ values vary from 0.46 to 0.79. However, Aquifer-2 head was much less affected by rainfall—the R^2^ values range from 0.30 to 0.59.

Figure 4c shows that monthly mean measured Kushabhadra River stages strongly affected monitored Aquifer-1 heads. River stage at Balianta affected Aquifer-1 heads with R^2^ values ranging from 0.46 (site O-1) to 0.71 (site O-16). River stage at Nimapara affected head with R^2^ values of 0.49 (site O-15) to 0.74 (site O-7). However, the river stage has slight to no influence on Aquifer-2 groundwater levels, with R^2^ varying from 0.03 to 0.39.

In summary, recharge from rainfall and recharge from the Kushabhadra River significantly contribute to the groundwater in an unconfined aquifer (Aquifer-1). Rainfall and the river contribute much less water to Aquifer-2.

### 3.2. Groundwater-Flow Simulation Results Computed by MODFLOW-2005

The accuracy of the model was graphically examined by the simultaneous plots of simulated and observed groundwater levels while using the pooled data (for each well and in each stress period) of groundwater levels at 13 shallow wells (Figure 5a) and 11 deep wells (Figure 5b). The correlation coefficient values of 0.89 (unconfined aquifer) and 0.87 (confined aquifer) signify good correlation between the simulated and observed groundwater levels. Comparing simulated versus observed hydrographs at individual wells shows that the simulation model represents groundwater level trends well at all sites, validating the model’s prediction ability [62].

Ref. [62] reported the calibration of the groundwater-flow simulation model. Both statistical and graphical indicators suggested a reasonably good calibration. All of the calibrated aquifer property values are within the ranges of observed field values [62]. The calibrated hydraulic conductivity values vary from 8.3 to 48.6 m/day in Aquifer-1, and from 13 to 85 m/day in Aquifer-2. Calibrated Aquifer-1 specific yield ranges from 0.03 to 0.23 and Aquifer-2 storage coefficient values vary from 1.46 × 10^−6^ to 7.76 × 10^−4^. Calibrated aquifer recharge ranges from 213.7 to 333.7 mm/year. Simulated Aquifer-1 groundwater-levels for pre-monsoon and post-monsoon seasons indicated that the groundwater flow direction in the unconfined aquifer is from north to east in the northern portion of the study area, and from north to south in the southern part of the study area. The groundwater flow direction in the confined aquifer (Aquifer-2) is from north to south. Sensitivity analysis revealed that model outputs are the most sensitive to changes in the confined aquifer hydraulic conductivity, followed by unconfined aquifer hydraulic conductivity, semi-confining layer vertical hydraulic conductivity, unconfined aquifer specific yield, confined aquifer storativity, and unconfined aquifer recharge [62].

The recharge from rainfall considerably contributes to area groundwater resources. Most of the 4.41 Mm^3^ recharge from the Kushabhadra and Bhargavi Rivers to Aquifer-1 occurs during the post-monsoon season. Groundwater discharge to rivers occurs as baseflow, mostly within the middle and downstream river reaches—0.85 Mm^3^ and 1.12 Mm^3^ during the pre-monsoon season and post-monsoon season, respectively.

### 3.3. Maximum Groundwater Abstraction Strategy Computed by S-OGM

**Figure 6:**
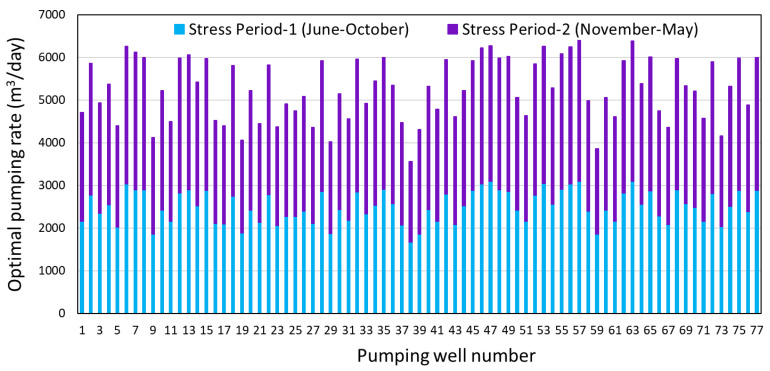
Monsoon season (Stress Period-1) and non-monsoon season (Stress Period-2) optimal pumping rates for the existing 77 wells in 2009, obtained from S-OGM.

The objective function values computed by S-OGM are the maximum total seasonal extraction rates from 77 existing wells that satisfy constraints and will not cause salt-water intrusion into aquifers near the coast. For 2009 conditions, these totals are 19.25 × 10^6^ m^3^ during the monsoon season (Stress Period-1) and 37.22 × 10^6^ m^3^ during the non-monsoon season (Stress Period-2). Figure 6 shows both seasonal rates for each well. The non-monsoon period’s pumping rate of each well is larger than the monsoon period’s pumping rate.

**Figure 7:**
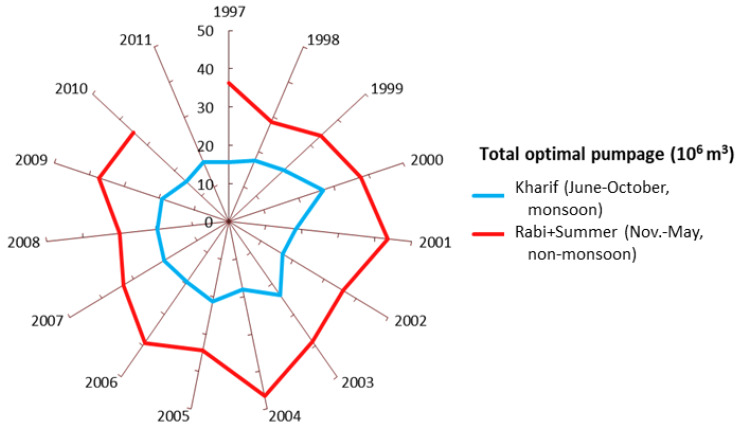
Seasonal total optimal groundwater withdrawals for 1997 to 2011, computed by S-OGM.

Figure 7 shows the total optimal seasonal groundwater withdrawals for the conditions of 1997–2011 computed by S-OGM. The greatest total non-monsoon extraction (46.5 × 10^6^ m^3^) occurs during 2004, followed by 2001 and 2006. During the monsoon season, the greatest total abstraction (27 × 10^6^ m^3^) occurs in 2000, followed by 2003 and 2005.

The hydraulic head values computed by S-OGM to occur at the end of each season satisfy the limits imposed within the maximum pumping optimization problem. Table 2 shows that all of the heads are above the −1 m lower limit needed to prevent seawater intrusion. Because groundwater pumping is greater during the non-monsoon period (Stress Period-2); heads at the end of Stress Period-2 are lower than the heads at the end of Stress Period-1.

It is worth mentioning that the groundwater pumping maximization problem is designed in order to determine the maximum simultaneous pumping at all wells that will not induce salt-water to intrude into the aquifer. Determining the actual optimal seasonal pumping from each well depends upon the cropping pattern for that season. Consequently, it is necessary to use the Resource Optimization Model (ROM) to determine the optimal cropping pattern and groundwater pumping rates needed to maximize net benefits from the available land and water resources.

### 3.4. Optimal Plan for Land and Water Resources Computed by ROM

The Resource Optimization Model (ROM) estimates a Rs. 3.7 billion total net annual income from the optimal cropping pattern proposed for 2009 conditions. Table 3 shows the contributing optimal allocation of individual crops to different land types, the optimal allocation of surface water and groundwater to different crops, and the net annual benefits from the optimal cropping patterns. The total gross annual irrigation water requirement is the sum of the annual total of 487.58 × 10^6^ m^3^–172.05 × 10^6^ m^3^ during the *Kharif* (monsoon) season and 315.52 × 10^6^ m^3^ in the *Rabi plus Summer* season. Much more rice area is assigned for the *Kharif* season than for the *Rabi* season, because the rice irrigation requirement per unit area is greater during the non-monsoon (*Rabi*) season. The *Rabi* season has more area being allocated to groundnut, green gram, and other vegetables that require less water than rice.

**Figure 8:**
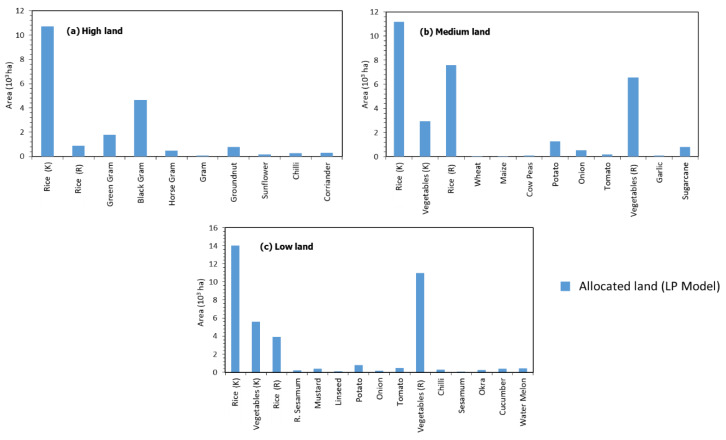
Comparison of cropping pattern obtained from ROM (Resource Optimization Model) by linear programming (LP) for: (**a**) high land, (**b**) medium land, and (**c**) low land. ‘K’ refers to the *Kharif* season and ‘R’ refers to the *Rabi* season.

Figure 8a–c show the optimal allocation of available land (high land, medium land, and low land) to different crops grown in *Kharif*, *Rabi*, and *Summer* seasons. These figures reaffirm that the ROM (Resource Optimization Model), solved by LP, allocates more of the available land to rice during the *Kharif* season when the southwest monsoon brings rain and reduces dependence on groundwater. During the *Rabi* season, ROM emphasizes allocation to crops having low water demand and providing greater net income (vegetables, green gram, black gram, horse gram, groundnut, mustard, garlic, and sunflower). For high land, ROM allocates more area to leguminous crops (green gram, black gram, and horse gram) than to rice (Figure 8a, *Rabi* season). In medium land and low land, more total area is allocated to vegetables, potatoes, and sugarcane than to rice (Figure 8b,c, *Rabi* season).

These findings suggest that the ROM provides reasonable results in terms of optimal annual income and the utilization of available land and water resources. Rice requires more water than other crops, and produces less profit per unit area than some other crops. Thus, the ROM suggests reducing the area currently used for rice cultivation and increasing areas for other suitable crops.

In other words, rice cultivation should be minimized, especially during the *Rabi* season, and crop diversification should be encouraged. These actions will enhance annual income and conserve water resources.

Furthermore, using model results to aid water use decision-making can help assure farmers of a dependable supply of irrigation water throughout the year. This will enable farmers to adopt more sustainable cropping systems and to grow crops and vegetables throughout the year without sacrificing profit or livelihood. Farmers’ livelihoods will improve by improving the sustainable utilization of basin’s land and water resources.

## 4. Conclusions

A numerical groundwater-flow simulation model for the Kushabhadra–Bhargavi River Delta of Eastern India was applied to quantitatively evaluate the connection between groundwater pumping rates and groundwater levels during pre-monsoon and post-monsoon seasons. By using convolution techniques to link the simulation model with an optimization algorithm, a resulting simulation-optimization (S-O) model used linear programming (LP) optimization to solve groundwater hydraulic optimization management problems. For the monsoon and non-monsoon seasons of 1997–2011, the S-O groundwater management model determined the optimal groundwater extraction patterns and maximum pumping rates from the existing irrigation wells that would satisfy specified management constraints. One constraint assured that any increased groundwater extraction would not induce saltwater intrusion into the aquifer from the ocean.

The computed maximum permissible seasonal groundwater withdrawal from the existing pumping wells (46.5 × 10^6^ m^3^) was for the non-monsoon season of 2004, followed by the non-monsoon seasons of 2001 and 2006. For monsoon seasons, the greatest computed groundwater extraction was 27 × 10^6^ m^3^ for year 2000, followed by 2003 and 2005. In other words, the maximum permissible seasonal groundwater abstractions were higher during the non-monsoon months of November through May, than through the monsoon months of June–October. The computed maximum pumping abstraction values were used as upper limits in the second optimization model.

Another optimization model was formulated to determine the optimal seasonal cropping patterns and associated water allocations, which would maximize the basin’s net annual economic return, subject to suitable land and water related constraints. This resource optimization model considered the conjunctive use of surface water and groundwater resources, the suitability of available cultivable land, and the types of crops historically grown in the basin. By using LP, this optimization model provides reasonable results in terms of annual income and utilization of available land and water resources. The optimal cropping patterns of the non-monsoon *Rabi* and *Summer* seasons have larger gross irrigation requirements than those of monsoon *Kharif* season. For 2009, the total annual gross irrigation requirement was estimated at 487.58 × 10^6^ m^3^.

Assuming 2009 conditions and unit costs and benefits, the optimal net annual economic return from the optimal cropping patterns and optimal water allocations are estimated to be Rs. 3.7 billion. The resource optimization model results suggest a reduction in the area that is allocated to rice and an increase in the area allocated to leguminous crops, sugarcane, potato, vegetables in the non-monsoon *Rabi* season. Rice cultivation consumes more water and provides less economic benefit per unit area. Reducing the *Rabi* season rice and diversifying crops would conserve vital natural resources and increase economic benefit.

In summary, the developed integrated simulation-optimization modeling framework and approach can aid the long-term planning and management of water resources (surface water and groundwater). The approach helps devise best management strategies for the conjunctive utilization of groundwater and surface water resources. Such scientific approaches help fill the gap between complex water resources modelling and real-world decision making. The demonstrated methodology can be replicated elsewhere for developing efficient groundwater management strategies to ensure groundwater sustainability under changing climatic conditions.

## Figures and Tables

**Table 1:** Area covered and Gross Irrigation Requirement for the crops grown in *Kharif*, *Rabi*, and *Summer* cropping seasons.

Crop Growing Season (Period)	Type of Crop		Area Covered (ha)	Gross Irrigation Requirement (m)
***Kharif* Season** (mid-June to October-end)	Paddy		22,893.37	0.36
	Vegetables		4973.34	0.48
***Rabi* Season** (November to February-end)	**Cereals**	Paddy	2919.8	1.44
		Wheat	28.34	0.456
		Maize	16.34	0.66
	**Pulses**	Green Gram	5870.1	0.18
		Black Gram	7773.6	0.18
		Horse Gram	640.2	0.3
		Gram	52.69	0.3
		Cow Peas	45.6	0.384
	**Oilseeds**	Groundnut	1788.4	0.42
		Sesamum	396.34	0.24
		Mustard	631.34	0.42
		Sunflower	177.52	0.36
		Linseed	105.6	0.24
	**Vegetables**	Potato	658.94	0.48
		Onion	115.36	0.72
		Tomato	267.7	0.48
		Other Vegetables	1812.3	0.42
	**Spices**	Chilli	247.3	0.312
		Garlic	71.08	0.54
		Coriander	274.23	0.36
		Sugarcane	376.9	2.04
***Summer* Season** (March to mid-June)	Sesamum		41.23	0.24
	Okra		85.7	0.48
	Cucumber		94.5	0.36
	Watermelon		95.6	0.54

**Table 2:** Optimal hydraulic heads (m MSL) at the head control locations.

Head Control Location	Aquifer Layer	Row	Column	Optimal Head (Stress Period-1)	Optimal Head (Stress Period-2)
1	Aquifer-1	17	13	12.78	12.3
2	Aquifer-2	42	44	12.57	12.25
3	Aquifer-2	44	51	10.26	9.87
4	Aquifer-2	43	51	12.15	11.78
5	Aquifer-1	45	31	12.14	11.67
6	Aquifer-2	49	17	6.25	5.87
7	Aquifer-1	50	17	12.01	11.78
8	Aquifer-1	53	45	8.15	8.02
9	Aquifer-1	58	54	9.68	9.23
10	Aquifer-2	58	55	10.8	8.55
11	Aquifer-1	65	48	8.18	7.45
12	Aquifer-1	16	24	8.45	7.56
13	Aquifer-1	68	40	11.15	10.8
14	Aquifer-1	79	33	11.25	10.5
15	Aquifer-1	81	24	8.09	7.6
16	Aquifer-2	64	24	9.45	8.65
17	Aquifer-2	50	41	4.41	4.2
18	Aquifer-2	17	40	5.06	4.5
19	Aquifer-2	19	41	6.43	5.87
20	Aquifer-1	23	23	7.53	6.45
21	Aquifer-2	32	20	5.03	4.5
22	Aquifer-1	33	47	4.17	4.05
23	Aquifer-1	37	23	5.08	4.6
24	Aquifer-2	41	41	8.45	7.65

**Table 3:** Optimal cropping patterns, gross irrigation requirement, and annual income computed by ROM.

Season	Crop	Optimally Allocated Area (ha)				Gross Irrigation Requirement (10^6^ m^3^)	Annual Income (10^7^ Rs.)
		High Land	Medium Land	Lowland	Total		
*Kharif*	Rice	11,231.52	11,167.37	14,031.2	36,430.09	131.15	92.90
	Vegetables		2933	5590	8523	40.91	71.93
*Rabi*	Rice	896	7590.8	3916	12,402.8	178.60	17.36
	Wheat	-	36.04	-	36.04	0.16	0.03
	Maize	-	24.54	-	24.54	0.16	0.02
	Green Gram	1785.1	-	-	1785.1	3.21	1.68
	Black Gram	4663.6	-	-	4663.6	8.39	6.72
	Horse Gram	485.2	-	-	485.2	1.46	0.03
	Gram	72.69		-	72.69	0.22	0.09
	Cow Peas	-	68.6	-	68.6	0.26	0.06
	Groundnut	794.4	-	-	794.4	3.34	1.15
	Sesamum	-	-	195	195	0.47	0.08
	Mustard	-	-	405	405	1.70	0.04
	Sunflower	192.2	-	-	192.2	0.69	0.50
	Linseed	-	-	105.6	105.6	0.25	0.01
	Potato	-	1258.94	781.84	2040.78	9.80	8.12
	Onion	-	514.36	175.36	689.72	4.97	4.16
	Tomato	-	187.7	477.7	665.4	3.19	5.29
	Other Veg	-	6571.5	10,976.3	17,547.8	73.70	147.75
	Chilli	282.3	-	297.3	579.6	1.81	1.94
	Garlic	-	95.08	-	95.08	0.51	0.58
	Corriander	304.23	-	-	304.23	1.10	0.63
	Sugarcane	-	798.9	-	798.9	16.30	1.95
*Summer*	Sesamum	-	-	78.7	78.7	0.19	0.05
	Okra	-	-	258.5	258.5	1.24	0.22
	Cucumber	-	-	387.5	387.5	1.40	1.05
	Water melon	-	-	445.5	445.5	2.41	1.78
Total						487.58	366.13
